# An Examination of the Relationship between Urinary Neurotrophin Concentrations and Transcutaneous Electrical Nerve Stimulation (TENS) Used in Pediatric Overactive Bladder Therapy

**DOI:** 10.3390/jcm10143156

**Published:** 2021-07-17

**Authors:** Joanna Bagińska, Edyta Sadowska, Agata Korzeniecka-Kozerska

**Affiliations:** Department of Pediatrics and Nephrology, Medical University of Białystok, 17 Waszyngtona Str., 15-274 Białystok, Poland; iklinped@umb.edu.pl (E.S.); agatakozerska@poczta.onet.pl (A.K.-K.)

**Keywords:** neurotrophins, transcutaneous electrical nerve stimulation, overactive bladder

## Abstract

This article aims to explore changes in urinary concentrations of selected neurotrophins in the course of TENS therapy in children with overactive bladder (OAB). A two-group open-label prospective study was conducted. The intervention group comprised 30 children aged between 5 and 12 years old with OAB refractory to conservative therapy. They received 12 weeks of TENS therapy in a home setting. The urinary neurotrophins, NGF, BDNF, NT3, NT4, were measured by ELISA at baseline and at the end of the TENS therapy. Total urinary neurotrophins levels were standardized to mg of creatinine (Cr). We compared the results with the reference group of 30 participants with no symptoms of bladder overactivity. The results revealed that children with OAB both before and after TENS therapy had higher NGF, BDNF, and NT4 concentrations in total and after normalization to Cr than the reference group in contrast to NT3. The response to the therapy expressed as a decrease of urinary neurotrophins after TENS depended on the age and the presenting symptoms. In conclusion, children older than 8 years of age with complaints of daytime incontinence responded better to TENS.

## 1. Introduction

Urinary incontinence is a frequent problem in pediatrics with increasing evidence that this is the second most common chronic condition of childhood after the atopy/allergy complex with a great impact on quality of life [1]. Overactive bladder (OAB) is the main cause of wetting in children defined by the International Children’s Continence Society (ICCS) as urinary urgency usually accompanied by frequency and nocturia, with or without urinary incontinence, in the absence of a urinary tract infection or other obvious pathology [2]. Although OAB is a widespread problem, the pathophysiology is still unclear. It is likely multifactorial but increasing recent evidence supports a central role of disturbed brain and bladder connection [3,4]. Previously, everything was concentrated only around the bladder. It has become apparent that the management of this problem should be based on the foundation that this is a delay in the children’s nervous system maturation. The available data indicate that pediatric OAB syndrome and many voiding dysfunctions may be part of a more generalized problem that affects multiple systems, notably, the bladder, bowels, and nervous system [5]. Several neurotransmitters have been identified in afferent nerves, including the family of neurotrophins: nerve growth factor receptors (NGF), brain derived neurotrophic factor (BDNF), neurotrophin-3 (NT3), and neurotrophin-4 (NT4). They play an important role in the plasticity of afferent nerves and spinal micturition pathways controlling bladder function [6], but the relationships between them and OAB have not been thoroughly investigated.

Most studies have compared urinary neurotrophin levels in OAB before and after pharmacological treatment. Antimuscarinic agents are currently the mainstay of OAB therapy. The reported rate of response is high and equals 70% to 80%, but the rate of complete symptom resolution is much lower and ranges from 14% to 35%. Urinary NGF and BDNF constitute the most well-studied neurotrophins as predictive biomarkers to assess the therapeutic outcome of antimuscarinic treatment. In the last decade, several studies on NGF were conducted in the adult population [7,8,9] with the same conclusions that urinary NGF levels are higher in patients with OAB when compared with a reference group, and decreased in response to anticholinergics. Antunes-Lopes et al. [10] found that urinary BDNF was increased in OAB patients at the baseline and were highly sensitive to OAB treatment with antimuscarinic agents. In addition, a strong correlation was found between the decrease of urinary BDNF concentration and the reduction in the number of urgency episodes per week. Several papers [11,12,13] on the pediatric population have appeared in recent years confirming previous observations that urinary NGF and BDNF could not only be potential biomarkers for children with OAB but also predictors of therapeutic efficacy. Interestingly, urinary levels of NGF and BDNF decreased upon OAB management, including not only pharmacological treatment but also lifestyle interventions [10] and detrusor injection of botulinum toxin A [14].

With the progress of medicine, novel efficacious options have emerged in the last decade in the management of OAB. Transcutaneous electrical nerve stimulation (TENS) is one of them and has become a subject of great interest [15]. TENS is based on neuromodulation, a process which regulates nervous system activity by controlling the physiological levels of neurotransmitters. Surprisingly, to the best of our knowledge, the relationship between neurotrophins and TENS efficacy in OAB has not been described. TENS has been used for pain control for several decades and provides relief to a remarkable number of patients affected by chronic pain. However, its value has been expanding in recent times due to rapid improvements in neuromodulation technologies, as well as research revealing more potential uses for this form of treatment. TENS has shown to be efficacious and has become a new tool in lower urinary tract dysfunction therapies [16]. According to the ICCS guidelines, the main indication for TENS in pediatrics is OAB refractory to the first line of treatment.

The exact mechanism of TENS action is unknown. There is a clear need to fill this knowledge gap. We aimed to pursue investigations of the changes in urinary concentrations of neurotrophins in pediatric patients with refractory OAB during TENS therapy. Based on the evidence available, we are not able to completely understand the pathophysiology of OAB as well as the exact mechanism of TENS action. The objective of this paper was to find a relationship between neurotrophins and TENS, which may help us to better understand the role of the nervous system in the pathophysiologic mechanisms responsible for OAB in childhood and the neurochemical pathways connecting the brain and bladder.

To the best of our knowledge, our study represents the first investigation of the relationships between TENS therapy and neurotrophins. The role of neurotransmitters excreted in the urine as biomarkers of the effectiveness of TENS therapy in children reporting OAB symptoms has not been described. The research at hand aimed to systematically describing how TENS influences the concentrations of neurotrophins and the clinical outcome in children with OAB. The detailed research goals included:

**Research** **Objective** **1.** To compare urinary neurotrophin concentrations between children with OAB and healthy participants.

**Hypothesis** **1** **(H1).** *The urinary level of neurotrophins will be elevated in children with OAB compared with healthy participants.*

**Research** **Objective** **2.** To establish the effects of TENS therapy on urinary concentrations of neurotrophins in the management of refractory OAB in children.

**Hypothesis** **2** **(H2).** *TENS therapy will result in decreasing urinary concentration of neurotrophins in children with refractory OAB.*

## 2. Materials and Methods

### 2.1. Participants

The open-label prospective study was conducted in 60 children (30 with OAB and 30 controls) aged between 5 and 17 years old between September 2020 and May 2021. To be eligible to enter the study, participants met the inclusion criteria presented in detail in Table 1. All 30 children with OAB had discontinued medical treatment with anticholinergics due to side effects or inefficacy and were off medical treatment for at least three months. Participants to the reference group were recruited to the study as children-volunteers of the hospital staff.

### 2.2. Intervention—TENS Treatment and Follow-Up Assessment

The intervention group received 12 weeks of TENS therapy in a home setting with a control visit after 4 and 8 weeks. Prior to starting the TENS therapy, the children in collaboration with their caregivers provided their medical history and underwent physical examination. Then they were thoroughly instructed on how to handle the TENS stimulator at home. A double-channel stimulator (Premier Stim Plus DIGITAL EM-6300) was used with 50 × 50 mm adhesive electrodes placed on the skin at the level of S2 to S3. A 2 Hz frequency was used with a 150 s pulse duration. The children were instructed to use the highest tolerable intensity up to a maximum of 40 mA. Sessions were performed once a day, every day for 2 h. During each follow-up, bladder diary, 48 h volume, and frequency charts were analyzed. After 12 weeks of treatment, TENS was discontinued in all patients. Throughout the study, the treatment was well-tolerated and no adverse events were reported.

### 2.3. Biochemistry

Urine samples of 10 mL were obtained as free-voided samples at the beginning and at the end of the TENS therapy in the intervention group and once in the reference group. Voided urine was put on ice immediately and transferred to the laboratory for preparation. The samples were centrifuged at 4000 rpm for 10 min at 4 °C. The supernatant was separated into aliquots in 2 mL tubes and preserved at −80 °C until measurements. Another 2 mL urine was stored separately to measure creatinine (Cr) levels.

NGF, BDNF, NT3, NT4 concentrations were determined using the immunoassay system for NGF (SEA105Hu, Cloud-Clone Corp., Wuhan, China), BDNF (SEA011Hu, Cloud-Clone Corp., Wuhan, China), NT3 (SEA106Hu, Cloud-Clone Corp., Wuhan, China), NT4 (SEA107Hu, Cloud-Clone Corp., Wuhan, China) with a specific and highly sensitive ELISA kit, which had a minimum sensitivity of 5.4 pg/mL for NGF, 11.6 pg/mL for BDNF, 5.7 pg/mL for NT3, and 6.0 pg/mL for NT4. Assays were performed according to the manufacturer’s instructions. All kit components and samples were brought to room temperature. Then, 100 μL of NGF/BDNF/NT3/NT4 standards, blank and urine was added to the appropriate wells, covered, and incubated for 1 h at 37 °C. Next, 100 μL of biotin antibody was added to each well, covered, and incubated for 1 h at 37 °C. After incubation, plates was washed three times and 100 μL of streptavidin solution was added to each well. The plate was incubated for 30 min at 37 °C and after incubation was washed five times. Then, tetra methyl benzidine (TMB) reactive substrate was added to the wells and incubated for about 30 min until the color development was visualized. Then, a stop solution was added and the plates were read at 450 nm with an ELISA reader.

The concentration of urinary Cr was measured using the calorimetric method by Jaffe (Cobas 6000 C501). The results were expressed as the total concentration of measured urine markers in pg/mL and were also divided by urinary Cr (mg/mL) for standardization as NGF/Cr, BDNF/Cr, NT3/Cr, NT4/Cr ratios.

### 2.4. Statistics

The data were collected in a Microsoft Excel database. Statistical analysis was performed using Statistica 13.0 (StatSoft Inc, Tulsa, OK, USA). Continuous variables were expressed as median and range, unless stated otherwise. All studied parameters were analyzed using nonparametric tests: Mann–Whitney, Kruskal–Wallis and chi-squared test. Correlations were assessed with the Spearman test. Values of *p* < 0.05 were considered significant.

### 2.5. Ethical Issues

This study was approved by the Ethics Committee of the Medical University of Bialystok (R-I-002/602/2019) which complies with the World Medical Association Declaration of Helsinki regarding ethical conduct of research involving human subjects and/or animals. All participants were given detailed information about the content of the study and all caregivers signed an informed consent form before initiation of the study.

## 3. Results

**Figure 1:**
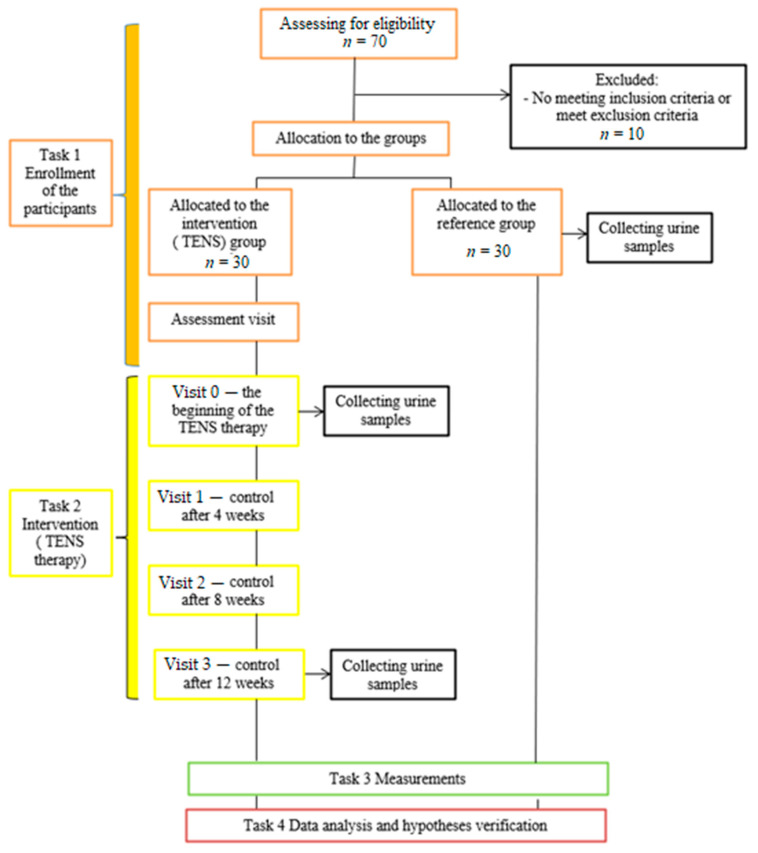
Outline of the organization of the study.

A detailed outline of the organization of the study is presented in Figure 1. We screened 70 participants: 40 to the study group and 30 to the reference group. We excluded 10 patients because they did not have OAB proven by urodynamic study, the diagnosis was made only on the basis of the patient’s symptoms. 

The patients’ clinical characteristics are presented in Table 2. There were no differences in the age, weight, height, or sex between the study and the reference group.

In comparison between the groups before and after TENS therapy, statistically significant differences were observed in the parameters obtained from bladder diaries and volume/frequency charts including the number of wet nights during a 30-day period with a median of 8 (0–30) before TENS in contrast to 3.5 (0–30) after TENS, the voiding frequency from 7.5 (5–23) to 5 (4–9) and minimum voided volume from 50 mL (15–130) to 80 mL (25–250) (*p* = 0.041, *p* = 0.029, *p* = 0.022, respectively; *p* < 0.05). No statistically significant differences were noted in maximal voided volume with a median of 195 mL (95–500 mL) before TENS and 190 mL (90–370 mL) after therapy (*p* = 0.53; *p* < 0.05).

**Figure 2:**
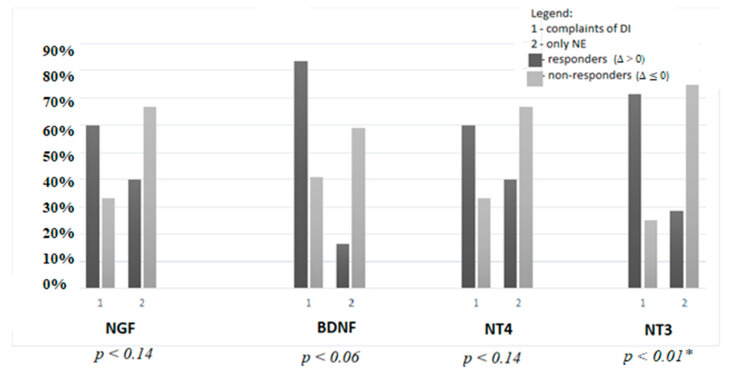
The comparison between response to the TENS therapy expressed as the change of urinary concentrations of neurotrophins in terms of urinary incontinence complaints. DI, daytime incontinence; NE, nocturnal enuresis, * *p* < 0.05.

The comparison of urinary neurotrophins revealed that children with OAB both before and after TENS therapy had higher NGF, BDNF, and NT4 concentrations in total and after normalization to Cr than the reference group, in contrast to NT3, which in total was lower in the study group but after normalization to Cr showed the same tendency. The differences are graphically presented in Figure 2. Additionally, a statistically significant difference was observed in urinary Cr.

To establish the effects of TENS therapy on urinary concentrations of neurotrophins, we calculated the “delta” values (Δ) for each neurotrophin. The Δ was considered as the changes in the absolute and ratios values of neurotrophins (subtracting the absolute value at baseline from the value at the end of TENS therapy). A Δ > 0 was considered a response to the therapy, while Δ ≤ 0 indicated the failure of the therapy. The Δ values are presented in detail in Table 3.

Only in the case of NGF the median Δ was higher than 0. Statistically significant differences were observed in urinary NGF according to age. The median age in children with Δ > 0 was 10.1 years (5.5–12.3) in contrast to 7.8 years (5.3–11) in children with Δ ≤ 0. We divided the cases into two subgroups—children older than 8 years at baseline and children aged 8 years or younger—and compared the response to TENS therapy. Twelve out of 18 (66.7%) children older than 8 years old responded well to the TENS therapy in contrast to 3/12 (25%) children younger than 8 years old. The differences were statistically significant (χ^2^ = 5.0, *p* = 0,025; *p* < 0.05). 

Moreover, children with complaints of daytime incontinence (DI) had a better response to TENS therapy in contrast to those presenting only with nocturnal enuresis (NE). Differences were noted in the urinary concentrations of all neurotrophins: NGF, BDNF, NT4, and NT3, but only in the case of the last one they were statistically significant (χ^2^ = 2.14, *p* = 0.14; χ^2^ = 3.39, *p* = 0.06; χ^2^ = 2.14, *p* = 0.14; χ^2^ = 6.47, *p* = 0.01, respectively), presented in detail in Figure 2.

## 4. Discussion

To the best of our knowledge, this is the first prospective study evaluating the efficacy of TENS in pediatric OAB with measurement of urinary neurotrophins. A homogeneous sample was chosen, comprising of patients only with confirmed detrusor overactivity in urodynamic examination without pharmacological treatment for at least three months before the intervention.

The literature on the efficacy of neuromodulation techniques in pediatric OAB shows a variety of approaches. At the beginning of the 2000s, the first studies of TENS in the treatment of urinary incontinence in children were done [17,18,19]. The pilot study was performed by Hoebeke et al. [17] in a group of children with urodynamically proven detrusor overactivity presenting with only DI with promising results, including an increase in cystometric bladder capacity, decrease in urge and incontinence episodes. Similar results were obtained by Bower et al. [18] with the first attempt to assess TENS therapy performed in a home setting. Malm-Buatsi et al. [19] reported a 73% urinary incontinence improvement after TENS therapy in OAB. A key limitation of all the above-mentioned research was that TENS was not the single therapy, the majority of participants continued taking anticholinergics. In the last decade, several controlled studies [20,21,22] have been conducted. Hagstroem et al. [20] conducted a study in which 27 children with refractory OAB were treated with TENS or sham stimulation for 4 weeks, with the conclusion that TENS had a superior effect to sham treatment, although the percent of responders in the active group was lower than reported previously in uncontrolled research. On the other hand, the results obtained from other controlled research suggested that the effect of neuromodulation in patients receiving urotherapy is marginal [21] and may give a high placebo effect based on the attention given to the child’s problem by the caregivers and physicians [22]. Such contradictory results show a need to find a better tool to answer the question of whether TENS is effective or not.

Our observations showed a reduction in the number of wet nights and frequency after TENS therapy. Additionally, an increase of minimum voided volume was recorded. However, the minimum storage after neuromodulation continued to be significantly lower than would be expected for children with no urinary problems. In contrast to some reports [17,18,23], we did not find statistically significant differences in maximal voided volume. It has been hypothesized that an increase in the bladder reservoir is the main mechanism of TENS action. Studies that evaluated the urodynamic parameters immediately after the first session of TENS as well as at the end of the therapeutic period demonstrated that the only urodynamic finding that showed improvement was bladder capacity [24]. Further studies [20,21] provided contradictory results, suggesting that the positive effect of TENS is the result of improved bladder sensation signals. Therefore, a new, reliable, and objective tool for evaluating TENS efficacy and relevant therapeutic outcomes is needed. It is well-described that the electric current stimulates the release of a wide range of neurotransmitters [6,25]. We wanted to take into account a novel theory and check changes in neurotrophins as a potential explanation for the TENS mechanism of action.

### 4.1. Neurotrophins at Baseline

NGF is the most studied and the best characterized neurotrophin in OAB, especially in the adult population [26]. Nonetheless, few studies have focused on the association between urinary NGF concentrations and OAB symptoms in children. According to the latest systemic review about the role of urinary NGF in pediatric OAB, three case-control studies were published between 2012 and 2017, which included 99 patients and 61 controls [27]. Children with OAB had a significantly higher baseline urinary NGF/Cr compared with controls, which is in accordance with our observations. However, the selected samples were heterogeneous. In 2012, Oktar et al. [11], as one of the first authors to do so, performed a study about the relation between pediatric OAB and NGF in the course of the anticholinergic treatment. However, not all participants had urodynamically confirmed detrusor overactivity. Additionally, the study group comprised patients with primarily diagnosed OAB but also those who had failed antimuscarinic treatment. The results indicated that, following antimuscarinic treatment, urinary NGF/Cr levels were significantly reduced at 6 months but not at 3 months. In 2015, a second study in the pediatric population was conducted in our Department by Korzeniecka-Kozerska et al. [12], with the inclusion criteria of primary diagnosed OAB proven in urodynamics before pharmacological treatment. Our actual study in contrast to the above-mentioned one analyzed NGF concentrations in children with refractory OAB after pharmacological treatment with persistent urinary symptoms. In 2016, another modification was made by adding BDNF as a potential biomarker and the combination of two urinary neurotrophins was measured in 24 patients with newly diagnosed OAB confirmed by urodynamics [13]. In the course of pharmacological treatment, BDNF showed a statistically significant decrease at 3 and 6 months of therapy in contrast to NGF. The authors suggested the use of urinary NGF/Cr as a marker for OAB diagnosis, and BDNF/Cr to evaluate treatment efficacy. The sensitivity and specificity of urinary BDNF levels for OAB patients was significantly higher than for NGF.

In our study we also observed elevated levels of NGF and BDNF in OAB compared with the control group at baseline. On the basis of previous reports [28] we could speculate that the overexpression of neurotrophins in OAB may be due to its extensive production by the urothelium, suburothelium, and the smooth muscle of the urinary tract. It was proven that especially NGF plays a key role in the pathophysiology of OAB, most probably by changing the threshold for bladder sensory neuron response. The increased amount of neurotransmitters substance being released by the urothelium leads to sensory urgency, and ultimately reflex bladder hyperactivity, causing OAB symptoms. Local release of NGF and BDNF by the urothelium was widely proven. The originality of our findings lies in the fact that we also measured two novel members of the neurotrophin family: NT3 and NT4. To our knowledge, an eventual relationship between them and OAB is totally unknown. They were scarcely investigated only from the theoretical point of view. They represent master modulators of neural plasticity, both in the peripheral and central nervous system, comparably to the better-described NGF and BDNF [29]. The role of NT3 and NT4 in the bladder dysfunction has not been described. NGF and BDNF have been identified in the peripheral tissues of the bladder, including the urothelium and detrusor muscles [30]. It has been suggested that NT3 and NT4 might also be expressed in the bladder, although their exact role and location are unclear [31].

In this paper, we proposed measurements of urinary NT3 and NT4 as potential markers of OAB and/or efficacy of TENS therapy. Interestingly, NT4 levels showed the same tendency as NGF and BDNF. At baseline, the total concentration was higher in OAB children than in the controls, but the differences were not statistically significant. After normalization to Cr, the differences were much more noticeable. Interestingly, NT3 acted differently to the others neurotrophins. Total concentrations of NT3 before TENS therapy were lower in the intervention group compared with the controls; whereas, after normalization to Cr, they showed an inverse relation. With a lack of studies on NT3 and urinary dysfunction we may only speculate about the reason. We found data suggesting a role of NT3 in the pathophysiology of nerve functions in diabetic animals with the conclusion that it may play a role in underactive bladder as a consequence of diabetic cystopathy [32,33]. Other studies on neurogenic underactive bladder stressed the role of decreased levels of NGF and NT3 in bladder compartments and afferent nerves as the most important changes leading to detrusor underactivity [34].

We observed statistically significant differences in urinary Cr between the studied groups. Cr differences could not be explained by variations in age and sex distributions across groups. Typically, Cr is thought to be excreted at a normal and constant rate in healthy children, so it is mostly used as a factor for normalization. However, in some studies it was found that the concentration of urinary Cr was highly variable in the adult population with OAB [35]. We decided to concentrate mostly on total levels of neurotrophins in formulating our conclusions.

### 4.2. Neurotrophins after TENS Therapy

In the whole study group we did not record statistically significant differences in urinary neurotrophins after TENS treatment, except for BDNF concentration, which surprisingly increased. Similarly to previous research on neurotrophins in the course of anticholinergic treatment, we assumed that increases in urinary markers at baseline would significantly diminish following TENS. Nevertheless, the general tendency was that the median levels of neurotrophins increased after the 12 weeks of therapy, but in particular cases, we observed a variation of neurotrophin concentrations. Some of the participants responded well to the therapy in contrast to those who completely failed the treatment. We attempted to identify those who were more likely to benefit from TENS by calculating delta values for each.

Interestingly, a good response to therapy expressed as a decrease of urinary levels of neurotrophins after TENS was observed in children presenting with DI. This result is in accordance with previous studies [36]. In this paper, while we refer to earlier work, measurement of the primary outcome is different. Previously, a reduction of urinary incontinence episodes was considered to be the main effect parameter. We used urinary biomarkers as a more objective tool.

In our study, all children had confirmed detrusor overactivity in urodynamic studies. In the clinical manifestation, the majority of patients (54%) complained only about NE. We observed the clinical response to TENS as a reduction of wet nights after therapy. However, this was not supported by a significant decrease in urinary neurotrophins in all cases. In our observations, NE may be a predictor of a worse response to TENS therapy. This may be a possible explanation for the lack of significant differences in urinary neurotrophins after TENS in the whole group. Additionally, modification of the time of day for TENS therapy may be a potential issue. In our study, we allowed patients to use TENS at any convenient time. It is possible that TENS performed before going to sleep or during the night in children with NE may be more effective. To prove such a statement, controlled studies are needed in the future.

Several studies about TENS in enuresis were performed. In 2010, Lorento et al. [37] evaluated the effectiveness of TENS in the treatment of non-monosymptomatic NE with 43% of cases cured, but 32% showed no improvement at all. In 2013, a prospective randomized clinical trial was performed in a group of children with primary monosymptomatic enuresis [38] treated with behavioral therapy and/or TENS. A statistically significant reduction in wet nights was observed (49.5 and 31.2%, respectively, at the end of treatment), but no patient had complete resolution of symptoms. A randomized, double-blind, placebo-controlled study was performed by Jørgensen et al. [39] on a group of 47 children with monosymptomatic NE demonstrating that TENS did not lead to significant changes in nocturnal urine production on wet or dry nights, voided volumes, or voiding frequency. Recently, a study on predictors of outcome in children and adolescents with OAB treated with TENS was performed. The results suggest that NE was the only symptom associated with poor outcome. The authors also assessed age as a potential predictor of response to TENS therapy but no statistically significant differences were observed [40]. In our study, children who were older than 8 years of age responded better to therapy when the effect of TENS was expressed as a decrease in NGF concentration after therapy. By contrast, the effectiveness rate in children younger than 8 years of age was much lower. The most likely explanation is the fact that older children may be more determined and motivated to use TENS, as well as follow the instruction more accurately. However, it cannot be excluded that it may be due to the maturation of nervous system control of micturition in older children.

The present study should be considered a preliminary one, because the main limitation of the experimental results is the small study sample. It should also be added that the optimum number of sessions, ideal time of the day, and TENS duration, as well as its settings, have not been established yet.

## 5. Conclusions

To the best of our knowledge, this is the first study in which four urinary neurotrophins were used as potential predictors of TENS efficacy in pediatric OAB. To conclude, we would like to highlight the following facts:

1. Urinary NGF, BDNF, and NT4 were increased in OAB refractory to the standard treatment in contrast to NT3;
2. The variation of urinary neurotrophins in the course of TENS depended on the age and clinical manifestation of OAB.

## Figures and Tables

**Table 1:** Criteria for enrollment to the study.

	Inclusion Criteria	Exclusion Criteria
Study group	- Age 5–18 years old with a clinical diagnosis of OAB with urodynamically proven detrusor overactivity - Refractory to standard urotherapy and pharmacotherapy with anticholinergics for at least 3 months - Normal physical examination and unremarkable urinary tract ultrasound	- Neurogenic bladder dysfunction - Anatomical abnormalities of the urinary or gastrointestinal track - Prior urinary tract surgery - Constipation according to Rome III criteria - Recurrent UTI within the last 3 months - High bladder capacity (>150% of expected bladder capacity) - Pacemakers or implantable defibrillators - Current urinary tract or vaginal infection - Lack of child’s cooperation
Reference group	- Age 5–18 years old with no urinary and nervous system problems - Normal physical examination and unremarkable urinary tract ultrasound	- Anatomical abnormalities of the urinary or gastrointestinal track - Recurrent UTI within the last 3 months - Current urinary tract or vaginal infection

**Table 2:** Characteristics of studied patients and comparison between groups.

	OAB Patients			Group C: Reference Group	*p*-Value				
	Group A: Before TENS		Group B: After TENS		A&B	A&C		B&C	
Median, Range									
Girls/boys (*n*)	15/15			19/11	-		0.39		0.39
Age (years)	8.7 (5.25–12.3)		8.83 (5.5–12.6)	8.4 (5–17)	0.55		0.85		0.6
Body weight (kg)	29.5 (17–62)			31.5 (15.5–70)	-		0.55		0.55
Height (cm)	134 (110–158)			135 (104–174)	-		0.81		0.81
NGF (pg/mL)	16.7 (5.4–101.3)	17.9 (6.33–103)		12.7 (6.3–73.7)	0.98		0.14		0.04 *
BDNF (pg/mL)	12.7 (11.3–189.9)	24.6 (11.3–515.3)		12.7 (11.3–40.8)	0.03 *		0.01 *		<0.001 *
NT4 (pg/mL)	107.9 (6–1245)	107.9 (6–2095)		79.4 (6.0–1096)	0.71		0.94		0.52
NT3 (pg/mL)	87 (5.7–1854)	72 (15.9–2934)		216 (17.9–4483)	0.81		0.03 *		0.04 *
Urinary Cr (mg/mL)	0.39 (0.04–2.4)	0.13 (0.04–1.5)		1.29 (0.49–3.32)	0.049 *	<0.001 *		<0.001 *	
NGF/Cr (pg/mg)	55.9 (3.3–894.6)	126.5 (5.6–802.5)		7.8 (2.4–68.7)	0.36	<0.001 *		<0.001 *	
BDNF/Cr (pg/mg)	49.7 (4.7–536.7)	217.7 (8.7–5725)		11.2 (3.8–44.9)	0.004 *	<0.001 *		<0.001 *	
NT4/Cr (pg/mg)	171.8 (11.2–6316)	748.6 (5.7–3958)		52.8 (2.2–664)	0.11	<0.001 *		<0.001 *	
NT3/Cr (pg/mg)	379.6 (3.5–3387)	687.5 (10.9–3532)		132.6 (10.8–2312)	0.18	0.36		0.03 *	

OAB—overactive bladder, Cr—creatinine, * *p* < 0.05.

**Table 3:** The delta values for each neurotrophin.

The Δ Values (Mean; Median, Range)		Responders/Non-Responders *n* (%)
NGF	4.38; 0.16 (−97.6–78.2)	15 (50)/15 (50)
BDNF	−45.8; −3.26 (−502–177.3)	7 (23)/23 (77)
NT4	−5.7; 2.63 (−2084–1201)	15 (50)/15 (50)
NT3	−6.97; −1.22 (−2840–1639)	14 (47)/16 (53)
NGF/Cr	30.7; 7.04 (−797–820)	12 (40)/18 (60)
BDNF/Cr	−567; −99 (−5693–219)	7 (23)/23 (77)
NT4/Cr	−173; −158 (−3939–5516)	11 (37)/19 (63)
NT3/Cr	−273; −59 (−2486–2293)	10 (33)/20 (67)

## Data Availability

The data presented in this study are available on request from the corresponding author. The data are not publicly available for ethical and privacy reasons.
